# CTLA4-Ig treatment induces M1–M2 shift in cultured monocyte-derived macrophages from healthy subjects and rheumatoid arthritis patients

**DOI:** 10.1186/s13075-021-02691-9

**Published:** 2021-12-24

**Authors:** Maurizio Cutolo, Stefano Soldano, Emanuele Gotelli, Paola Montagna, Rosanna Campitiello, Sabrina Paolino, Carmen Pizzorni, Alberto Sulli, Vanessa Smith, Samuele Tardito

**Affiliations:** 1 Laboratory of Experimental Rheumatology and Academic Division of Clinical Rheumatology, Department of Internal Medicine, IRCCS San Martino Polyclinic Hospital, University of Genova, Genoa, Italy; 2 Department of Rheumatology, Ghent University Hospital, Ghent, Belgium; 3 Department of Internal Medicine, Ghent University, Ghent, Belgium; 4 Unit for Molecular Immunology and Inflammation, VIB Inflammation Research Center (IRC), Ghent, Belgium

**Keywords:** CTLA4-Ig, Abatacept, Monocytes, Macrophages, Rheumatoid arthritis, M1–M2 polarisation, Autoimmune

## Abstract

**Background:**

In rheumatoid arthritis (RA), macrophages play an important role in modulating the immunoinflammatory response through their polarisation into “classically” (M1) or “alternatively activated” (M2) phenotypes. In RA, CTLA4-Ig (abatacept) reduces the inflammatory activity of macrophages by interacting with the costimulatory molecule CD86. The study aimed to investigate the efficacy of CTLA4-Ig treatment to induce an M2 phenotype both in M1-polarised monocyte-derived macrophages (MDMs) obtained from healthy subjects (HS) and in cultured MDMs obtained from active RA patients.

**Methods:**

Cultured MDMs were obtained from peripheral blood mononuclear cells of 7 active RA patients and from 10 HS after stimulation with phorbol myristate acetate (5 ng/mL) for 24 h. HS-MDMs were then stimulated with lipopolysaccharide (LPS, 1 mg/mL) for 4 h to induce M1-MDMs. M1-MDMs and RA-MDMs were treated with CTLA4-Ig (100 μM and 500 μM) for 3, 12, 24, and 48 h. The gene expression of CD80, CD86, and TLR4 (M1 markers); CD163, CD204, and CD206 (surface M2 markers); and MerTK (functional M2 marker) was evaluated by qRT-PCR. The protein synthesis of surface M2 markers was investigated by Western blotting. The statistical analysis was performed by the Wilcoxon *t*-test.

**Results:**

In LPS-induced HS-M1-MDMs, CTLA4-Ig 100 μM and 500 μM significantly downregulated the gene expression of M1 markers (3 h *p*<0.01 for all molecules; 12 h *p*<0.05 for TLR4 and CD86) and significantly upregulated that of M2 markers, primarily after 12 h of treatment (CD163: *p* < 0.01 and *p* < 0.05; CD206: *p* < 0.05 and *p* < 0.01; CD204: *p* < 0.05 by 100 mg/mL). Moreover, in these cells, CTLA4-Ig 500 μM increased the protein synthesis of surface M2 markers (*p* < 0.05). Similarly, in RA-MDMs, the CTLA4-Ig treatment significantly downregulated the gene expression of M1 markers at both concentrations primarily after 12 h (*p* < 0.05). Furthermore, both concentrations of CTLA4-Ig significantly upregulated the gene expression of CD206 (after 3 h of treatment; *p* < 0.05), CD163, and MerTK (after 12 h of treatment, *p* < 0.05), whereas CD204 gene expression was significantly upregulated by the high concentration of CTLA4-Ig (*p* < 0.05). The protein synthesis of all surface markers was increased primarily by CTLA4-Ig 500 μM, significantly for CD204 and CD206 after 24 h of treatment (*p* < 0.05).

**Conclusions:**

CTLA4-Ig treatment seems to induce the in vitro shift from M1 to M2 macrophages, of both HS-M1-MDMs and RA-MDMs, as observed by the significant downregulation exerted on selected M1 markers and the upregulation of selected M2 markers suggesting an additional mechanism for its modulation of the RA inflammatory process.

**Supplementary Information:**

The online version contains supplementary material available at 10.1186/s13075-021-02691-9.

## Introduction

Rheumatoid arthritis (RA) is a complex autoimmune disease characterised by chronic inflammation leading to progressive disability, mainly involving synovial joints, and affecting approximately 1% of the population worldwide [1–3]. The mechanisms underlying the inflammation in RA have been analysed in many immunological studies shedding light on the role of innate immunity in modulating the adaptive immunity, controlling the immune response at different levels and mediating the induction, as well as the progression, of persistent inflammation [4, 5].

Among the inflammatory cells involved in RA, macrophages potentially play a central pathogenic role contributing to the chronicity of the disease [4–8]. Macrophages invading the synovial tissue are stimulated to release cytokines, primarily tumour necrosis factor-α (TNFα) interleukin (IL)-1, IL-6, IL-17, and IL-23; chemotactic factors (such as macrophage chemoattractant protein-1); and matrix metalloproteinases (MMPs), resulting in bone and cartilage destruction [9, 10]. Local microenvironmental signals are crucial in macrophage activation having an effect on their plasticity through the promotion of a functional polarisation towards either classically activated (M1) or alternatively activated (M2) phenotype [7, 9, 11]. However, in the modulation of the immune response, M1 and M2 macrophages only appear to be two extremes in the dynamic change of the macrophage phenotype [7, 10–14].

M1 macrophages are defined by the expression of specific surface and cytoplasmatic markers, including Toll-like receptors (i.e. TLR2 and TLR4) and costimulatory molecules (i.e. cluster differentiation CD80 and CD86); they mainly secrete numerous pro-inflammatory cytokines such as TNFα, IL-1, and IL-6, which are responsible for joint damage [15–17].

Conversely, M2 macrophages exert anti-inflammatory effects and are phenotypically characterised by the expression of specific surface markers, including macrophage scavenger receptors (CD204 and CD163) and mannose receptor-1 (CD206), in addition to the release of IL-10 and transforming growth factor-b (TGF-β) [7, 11].

Interestingly, recent studies have found that the imbalance between M1 and M2 is correlated with the worsening of RA disease [10, 16, 18–20]. In addition, the increase in pro-inflammatory cytokines secreted by M1 macrophages contributes to exacerbate RA flares, whereas the release of anti-inflammatory cytokines mediated by M2 macrophages seems to reverse the inflammatory condition [21].

Wang et al. reported an imbalance in the M1/M2 macrophage ratio in RA patients, confirming a disequilibrium between these two phenotypes and suggesting its potential role in the pathogenesis of RA [16]. Among the molecules that promote macrophage polarisation towards one of the two functional states, some are of interest and need to be mentioned: granulocyte-macrophage colony-stimulating factor (GM-CSF), interferon-g (IFN-g), and lipopolysaccharide (LPS) induce the polarisation into a pro-inflammatory M1 phenotype, whereas macrophage colony-stimulating factor (M-CSF), IL-4, IL-10, and IL-13 promote the anti-inflammatory M2 phenotype [22–25]. Moreover, it has been demonstrated that anticitrullinated protein antibodies (ACPAs) activate IFN regulatory factor 5 (IRF5) inducing M1 macrophage polarisation, while IRF4 specifically promotes M2 macrophage polarisation [26].

Some studies reported how drugs may contribute to macrophage polarisation [11, 15, 26–28]. Currently, few studies are available on the ability of biological disease-modifying anti-rheumatic drugs (bDMARDs) to promote the polarisation, and recently, Degboe et al. demonstrated the efficacy of anti-TNFα agents in inhibiting in vitro macrophage inflammatory functions and favouring the resolution of inflammation through the polarisation towards alternative macrophage features [29].

Among the other known multiple bDMARDs used in the treatment of RA according to the EULAR guidelines, CTLA4-Ig fusion protein (CTLA4-Ig, abatacept) has proved to modulate the immune response thanks to its innovative pharmacodynamics [30–32]. This fusion protein combines the extracellular domain of the cytotoxic T lymphocyte-associated antigen-4 with the heavy chain fragment of immunoglobulin G, and it is used to treat several autoimmune inflammatory conditions, including RA, psoriatic arthritis, lupus erythematosus, and inflammatory bowel disease [32]. In RA patients who have an inadequate response to biologic DMARDs, CTLA4-Ig was recently observed to reduce the composite score of Disease Activity Score for 28 Joints based on the C-reactive protein level (DAS28/CRP) after 12 weeks of treatment, as well as the DAS28/CRP core components, such as tender joint count and swollen joint count of 28 joints, and the patient’s global assessment of disease activity [33]. Moreover, in these RA patients, CTLA4-IG confirmed its higher safety compared to other drugs, such as Janus kinase inhibitors [33]. In a recent study which investigated the histological and transcriptomic effect of CTLA4-Ig on the synovia of active RA patients despite methotrexate, it was observed that the treatment with this fusion protein significantly downregulated both myeloid leukocytes and T cell activation pathways [34].

In an insulin-resistant mouse model, CTLA4-Ig was demonstrated to contribute to the macrophage shift from M1 to M2, reducing adiponectin gene expression and alleviating adipose tissue inflammation through the suppression of TNFα and IL-6 expression [35].

Further observations revealed that the treatment with abatacept of cultured human synovial macrophages drastically reduced their production of pro-inflammatory cytokines, such as IL-1, IL-6, and TNFα, interfering with the activation of NF-kB transcription factor and suggesting its possible action directly on antigen-presenting cells, including macrophages themselves [36–38].

Based on these observations, the aim of the present study was to investigate the potential role of CTLA4-Ig to promote the shift from M1 to M2 in cultured human M1-polarised monocyte-derived macrophages (M1-MDMs) obtained from healthy subjects (HS) and RA patients, by evaluating the modulation of specific surface and functional markers.

## Materials and methods

### RA patients and HS enrolment

Seven RA patients (five females and two males, mean age 54 ± 13 years), who fulfilled the 2010 ACR/EULAR Classification Criteria for Rheumatoid Arthritis [39], and ten HS (two females and eight males, mean age 51 ± 14) were recruited at the Division of Rheumatology, Genova University.

Eligible patients were ≥ 18 years of age and had adult-onset RA for ≥ 3 months as defined by the 2010 American College of Rheumatology (ACR) criteria [39]. Present and recent constant uses of analgesics, nonsteroidal anti-inflammatory drugs (NSAIDs), low oral corticosteroids dose (less than 5 mg/day prednisone equivalent), and only conventional synthetic csDMARDs (low-dose methotrexate or leflunomide) were permitted for the 7 selected RA patients (Table 1).

**Table 1:** Demographic and clinical characteristic of the RA patients

**Demographic characteristics of the RA patients**	
Age (years, mean ± SD)	54 ± 13
Sex (female/male)	4/1
**Autoantibodies and RA patients with hand RX erosions**	
+RF (*n*/5 = %)	3 (60%)
+ACPA (*n*/5 = %)	3 (60%)
Rx erosions (*n*/5 = %)	3 (60%)
**Disease duration (years, mean ± SD)**	6.4 ± 5
0–5 years (*n*/5 = %)	3 (60%)
5–10 years (*n*/5 = %)	2 (40%)
> 20 years (*n* = %)	0 (0%)
**Comorbidities**	
Hypertension (*n*/5 = %)	3 (60%)
Diabetes (*n* = %)	0 (0%)
Anaemia (*n* = %)	0 (0%)
Sjogren’s syndrome (*n* = %)	0 (0%)
Heart failure (*n* = %)	0 (0%)
Acute cardiac ischemic (ACI) (*n* = %)	0 (0%)
Allergic asthma (*n* = %)	0 (0%)
Dyslipidaemia (*n*/5 = %)	1 (20%)
Neoplasia (*n* = %)	0 (0%)
Osteoporosis (*n*/5 = %)	3 (60%)
Dysthyroid (*n*/5 = %)	1 (20%)
*H. pylori* (*n* = %)	0 (0%)
HBV (*n* = %)	0 (0%)
**NSAID treatment**	
NSAIDs (*n*/5 = %)	1(20%)
**Glucocorticoid treatment**	
Prednisone (*n*/5 = %)	1 (20%)
Modified release prednisone (*n*/5 = %)	2 (40%)
**csDMARD treatment**	
Methotrexate (*n*/5 = %)	3 (60%)
Leflunomide (20 mg/day) (*n*/5 = %)	2 (30%)
**bDMARD treatment**	
TNF inhibitors (*n*/5 = %)	1 (20%)
Abatacept (*n* = %)	0 (0%)
Rituximab (*n* = %)	0 (0%)
Tocilizumab (*n* = %)	0 (0%)
**Other treatments**	
Vitamin D (*n*/5 = %)	4 (80%)
Folic acid supplementation (5 mg/week) (*n*/5 = %)	3 (60%)
**Clinical score and laboratory findings**	
Tj (mean ± SD)	5 ± 2.97
Sj (mean ± SD)	1.8 ± 1.47
VAS (mean ± SD)	5.2 ± 2.63
CRP (mg/dL, mean ± SD)	11.36 ± 16.47
DAS (CRP) (mean ± SD)	3.71 ± 0.56

*SD* standard deviation, *RF* rheumatoid factor, *ACPA* anticitrullinated protein antibodies, *H. pylori Helicobacter pylori*, *HBV* hepatitis B virus, *NSAIDs* nonsteroidal anti-inflammatory drug, *MR prednisone* modified release prednisone, *csDMARDs* conventional synthetic disease-modifying anti-rheumatic drugs, *bDMARDs* biological disease-modifying anti-rheumatic drugs, *TNF inhibitors* tumor necrosis factor inhibitors, *TJ* tender joints, *SJ* swollen joints, *VAS* visual analogue scale, *CRP* c-reactive protein, *DAS* disease activity score

All RA patients and HS provided informed consent, and the study was approved by the local ethics committee.

### Cell cultures and treatments

In order to test the most appropriate stimuli to induce the pro-inflammatory condition, cultured human monocytic leukaemia (THP1) cell line and circulating monocytes obtained from HS were activated to macrophages by phorbol myristate acetate (PMA, 5 ng/mL, Sigma-Aldrich, Milan, Italy) for 24 h and then treated with LPS (1 mg/mL) alone or in combination with INF-γ (20 ng/mL) (Sigma-Aldrich) for 4 h [22]. The quantitative real-time polymerase chain reaction (qRT-PCR) analysis demonstrated a clear reliable acquisition of an M1 inflammatory functional status of cultured THP1-derived macrophages and HS-MDMs, through the upregulation of the gene expression of TLR4, CD80, and CD86 (M1 phenotype markers) together with the downregulation of the gene expression of CD163, CD204, and CD206 (M2 phenotype markers), only after the stimulation with LPS, which therefore was used for the in vitro experiments to induce the M1 phenotype in MDMs (Figs. S1 and S2).

Cultured human monocytes were isolated from peripheral blood mononuclear cells (PBMCs) of HS and RA patients by density gradient centrifugation using Ficoll-Paque (Sigma-Aldrich) and overnight adhesion in tissue culture dishes in the presence of growth medium (RPMI at 10% of foetal bovine serum, 1% of penicillin-streptomycin, and 1% l-glutamine-Euroclone, Milan, Italy).

Cultured HS and RA monocytes were treated with PMA (5 ng/ml, Sigma-Aldrich) for 24 h to induce their differentiation into MDMs (HS-MDMs and RA-MDMs, respectively). At this step, cultured HS-MDMs were stimulated with LPS (1 mg/mL, Sigma-Aldrich) for 4 h to induce their polarisation into a pro-inflammatory M1 phenotype (HS-M1-MDMs) [22]. Afterwards, HS-M1-MDMs were treated with or without CTLA4-Ig at the concentrations of 100 μg/mL and 500 μg/mL for 3, 12, 24, and 48 h.

Similarly, cultured RA-MDMs were treated or without CTLA4-Ig (100 and 500 μg/ml) for 3, 12, 24, and 48 h. Cultured HSs-MDMs or RA-MDMs maintained in growth medium without any stimulation or treatment were used as unstimulated cells.

Based on our previous in vitro studies using different cell types, such as THP1-derived macrophages and RA synovial macrophages, the best modulatory effect of the treatment with CTLA4-Ig in one single administration on the gene expression was obtained earlier (3 and 12 h) rather than later on (24 and 48 h) [36, 40, 41].

### Quantitative real-time polymerase chain reaction (qRT-PCR)

Total RNA was extracted using the RNA/Protein Purification Plus Kit (Norgen Biotek Corp., Canada) or Nucleospin RNA/Protein kit (Macherey Nagel, Germany) based on the number of cultured cells. The RNA was quantified by Nanodrop (Thermo Fisher Scientific, MA, USA) which also allowed the evaluation of RNA integrity, and retrotranscribed (until 3μg) using Quantitec reverse transcription kit (QIAGEN, Netherlands) to obtain cDNA. The cDNA was amplified in a real-time experiment with Eppendorf Realplex 4 Mastercycler (Hamburg, Germany).

In order to select the most appropriate and stable housekeeping (HK) gene, four potential HK genes were selected: glyceraldehyde 3-phosphate dehydrogenase (GAPDH, NM_001256799.3), beta (b)-Actin (NM_001101.5), human ubiquitin-conjugated enzyme E2D2 (hUBE2D2, NM_003339.3), and ribosomal protein L13A (RPL13A, NM_012423.4) [42]. The stability of the chosen HK genes was determined in preliminary experiments in cultured THP1-derived macrophages reporting no significant variance, as well as in M1-MDMs, where the hUBE2D2 gene showed the best integrity and higher primer efficiency.

The gene expression of M1 macrophage markers (CD80, CD86 and TLR4), surface M2 macrophage markers (CD206, CD204, CD163), and a functional M2 marker proto-oncogene tyrosine-protein kinase MER (MerTK) was investigated using the following primers: CD80 = NM_005191, CD86 = NM_001206924, TLR4 = NM_003266, CD206 = NM_002438, CD204 = NM_001363744, CD163 = NM_001370145, and MerTK = NM_006343.3. Relative quantification of gene expression was performed using the 2^−ΔΔCT^ method [43]. The melting curve was included in all qRT-PCR assays to confirm the specificity of the SYBR green assay.

### Western blotting

Proteins were extracted in ice using radio-immunoprecipitation assay (RIPA) buffer: tris(hydroxymethyl)aminomethane-hydrochloric acid [TrisHCl] pH 7.4 25 mM, sodium chloride [NaCl_2_] 150 mM, Triton X-100 1%, sodium dodecyl sulphate [SDS] 0.1%, ethylene glycol-bis(β-aminoethyl ether)-N,N,N′,N′-tetraacetic acid [EGTA] and ethylenediaminetetraacetic acid [EDTA] 1 mM, and 1× protease inhibitors [sodium orthovanadate (Na_3_VO_4_), phenylmethylsulphonyl fluoride (PMSF), leupeptin, aprotinin; Sigma-Aldrich]. Proteins were quantified by the bicinchoninic acid (BCA) method.

For Western blotting assays, 60 μg of proteins was separated by electrophoresis on 4–12% gradient Tris-glycine gels (GenScript, NY, USA) and then transferred onto a Hybond-C-nitrocellulose membrane (Thermo Fisher Scientific).

After 1 h in blocking solution (PBS 1× and 0.1% Triton X-100) at 5% of non-fat powdered milk or 5% of bovine serum albumin (BSA), the membranes were incubated overnight at 4 °C with the primary antibodies anti-human CD204 (dilution 1:500; Abcam, Cambridge, UK), CD163, and CD206 (dilution 1:500; Cell Signalling Technology, Danvers, MA, USA).

The membranes were subsequently incubated with the following horseradish peroxidase (HRP)-conjugated secondary antibodies: anti-mouse IgG for CD204, anti-rabbit IgG for CD206, and CD163 (dilution 1:2000; Cell Signalling Technology).

The membranes were also incubated with primary HRP-conjugated antibodies to human GAPDH or β-actin (dilution 1:1000 and 1:2000, respectively; Santa Cruz Biotechnology, Dallas, USA) to confirm similar loading of protein samples and the efficacy of the electrophoretic transfer.

Protein synthesis was detected using the enhanced chemiluminescence system (SuperSignal™ West Femto Maximum Sensitivity Substrate, Thermo Fisher Scientific), and the related densitometric analysis was performed with the Uvitec Image Analysis System (Uvitec, Cambridge, UK). Western blotting was performed for each independent in vitro experiment with cultured M1-MDMs and RA-MDMs.

For each experimental condition, the value of the synthesis of CD204, CD206, CD163, TLR4, CD80, and CD86 was normalised to that of the corresponding GAPDH or β-actin. The resulting value of each treatment was compared with that of the related untreated cells (taken as unit value).

### Statistical analysis

The statistical analysis was carried out with GraphPad Prism (version 8.4.0, GraphPad Software, San Diego, CA, USA). Differences in continuous unpaired variables were tested using non-parametric Mann-Whitney *U*-test and Wilcoxon test for continuous paired variables. Any *p*-value lower than 0.05 was considered as statistically significant. The results of qRT-PCR and Western blotting are reported as mean ± standard deviation (SD).

The Spearman rank non-parametric correlation test was performed to investigate whether the downregulated gene expression of M1 markers or the upregulated gene and protein expression of M2 markers (dependent variable) and CTLA4-Ig dosage (independent variable) were related. To know the correlation strength, we use the following guideline: − 0.3 ≤ rho ≤ 0/0 ≤ rho ≤ + 0.3: “weak” association; − 0.3 ≤ rho ≤ − 0.7/+ 0.3 ≤ rho ≤ + 0.7: “moderate” association; and − 0.7 ≤ rho ≤ − 1/+ 0.7 ≤ rho ≤ + 1: “strong” association [44]. The more the rho’s value is close to + 1 or − 1, the more the association is considered monotonic [44, 45].

## Results

### Abatacept significantly downregulated the TLR4, CD80, and CD86 M1 markers and upregulated the CD163, CD204, and CD206 M2 markers in cultured HS-M1-MDMs

**Fig. 1:**
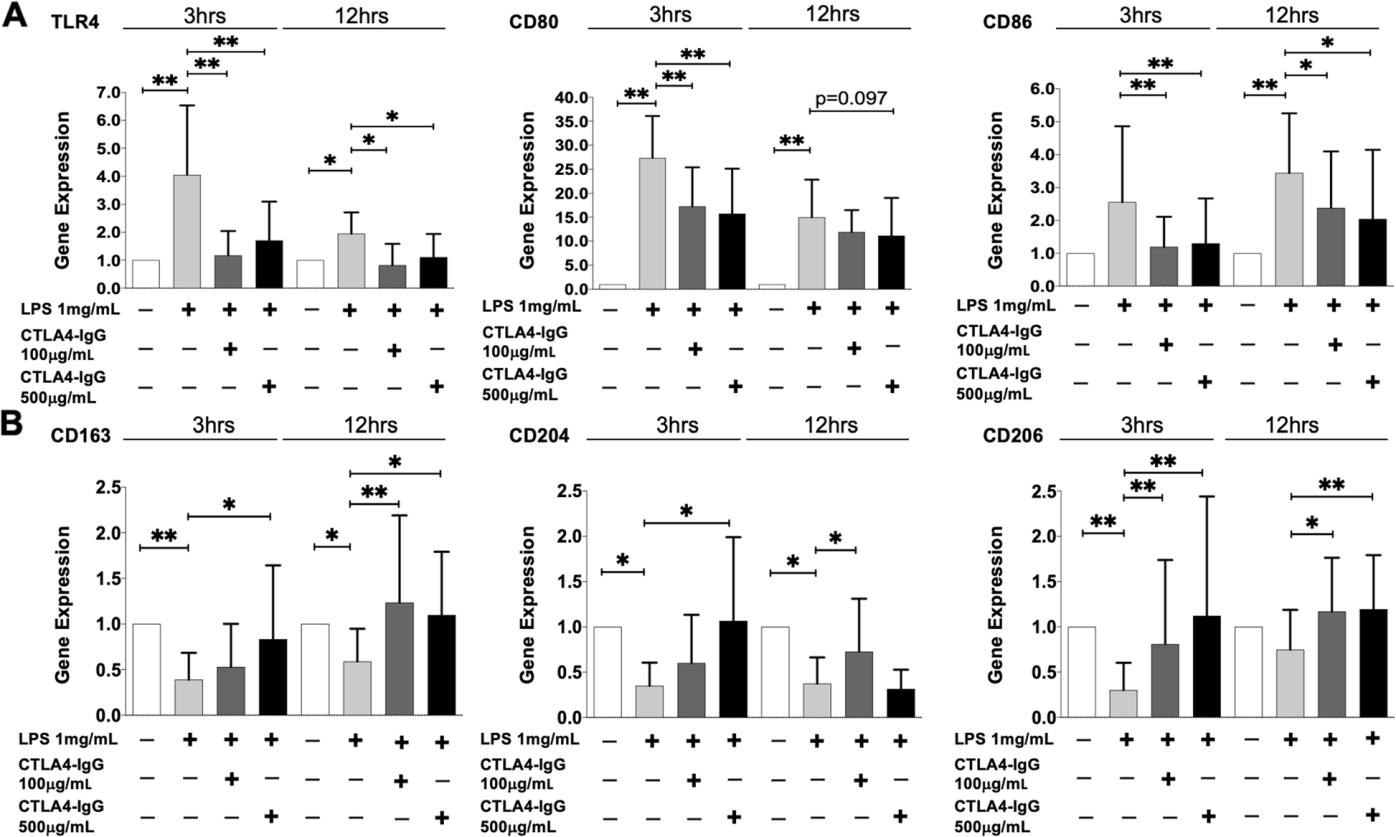
Gene expression of M1 and M2 markers in cultured HS-M1-MDMs treated with CTLA4-Ig. Quantitative real-time PCR of M1 markers (TLR4, CD80, CD86) or M2 markers (CD163, CD204, CD206) in cultured HS-MDMs maintained in normal growth medium without any stimulation or CTLA4-Ig treatment (white bar), stimulated with LPS alone (HS-M1-MDMs) (light grey bar), or stimulated with LPS and then treated with CTLA4-Ig (abatacept) at a concentration of 100 μg/mL (dark grey bar) and 500 μg/mL (black bar) for 3 and 12 h. **A** M1 markers (TLR4, CD80, CD86). **B** M2 markers (CD163, CD204, CD206). Final results were obtained from ten independent in vitro experiments. *p < 0.05; **p < 0.01

The stimulation with LPS induced cultured HS-MDMs to acquire an M1 macrophage phenotype (HS-M1-MDMs) through the upregulation of the gene expression of all the investigated M1 markers compared to unstimulated cells; this upregulation was significant for TLR4 and CD80 already after 3 h of stimulation (*p* < 0.01 for both markers; CD86 *p* = 0.18; Fig. 1A). The gene expression of all these M1 phenotype markers was maintained significantly upregulated by LPS also after 12 h of stimulation (*p* < 0.05 for TLR4, *p* < 0.01 for CD80 and CD86 vs. unstimulated cells; Fig. 1A). At the same time, LPS significantly downregulated the gene expression of all M2 phenotype markers compared to unstimulated MDMs (CD163: *p* < 0.01 at 3 h and *p* < 0.05 at 12 h; CD204: *p* < 0.05 both at 3 and 12 h; CD206: *p* < 0.01 at 3 h; Fig. 1B).

In cultured HS-M1-MDMs, CTLA4-Ig treatment at the concentrations of 100 μM and 500 μM significantly reduced the upregulation of the gene expression of all M1 markers induced by LPS already after 3 h of treatment (TLR4, CD80, CD86: *p* < 0.01 vs. untreated HS-M1-MDMs for both concentrations; Fig. 1A). The downregulatory effect of CTLA4-Ig treatment on the gene expression of M1 markers was maintained significant also after 12 h of treatment for TLR4 and CD86 compared to untreated HS-M1-MDMs (*p* < 0.05 for both concentrations; Fig. 1A).

Of note, in cultured HS-M1-MDMs, CTLA4-Ig (100 μM and 500 μM) upregulated the gene expression of all the investigated M2 markers after 3 and 12 h of treatment (Fig. 1B). In detail, CTLA4-Ig 100 μM upregulated the gene expression of CD163 and CD204 but significantly only that of CD206 after 3 h of treatment (*p* < 0.01 vs. untreated HS-M1-MDMs; Fig. 1B). Noteworthy, the gene expression of all these M2 phenotype markers was significantly increased by CTLA4-Ig 100 μM after 12 h of treatment (*p* < 0.01 for CD163, *p* < 0.05 for CD204 and CD206 vs. untreated HS-M1-MDMs; Fig. 1B). Instead, CTLA4-Ig 500 μM significantly upregulated the gene expression of all the investigated M2 phenotype markers in cultured HS-M1-MDMs already after 3 h of treatment (*p* < 0.05 for CD163 and CD204; *p* < 0.01 for CD206 vs. untreated HS-M1-MDMs; Fig. 1B). The increased gene expression of CD163 and CD206 was maintained statistically significant by CTLA4-Ig 500 μM after 12 h of treatment, compared to cultured untreated HS-M1-MDMs (*p* < 0.05 and *p* < 0.01, respectively; Fig. 1B).

In cultured HS-M1-MDMs, the modulation of these M2 phenotype markers was investigated also at the protein level.

**Fig. 2:**
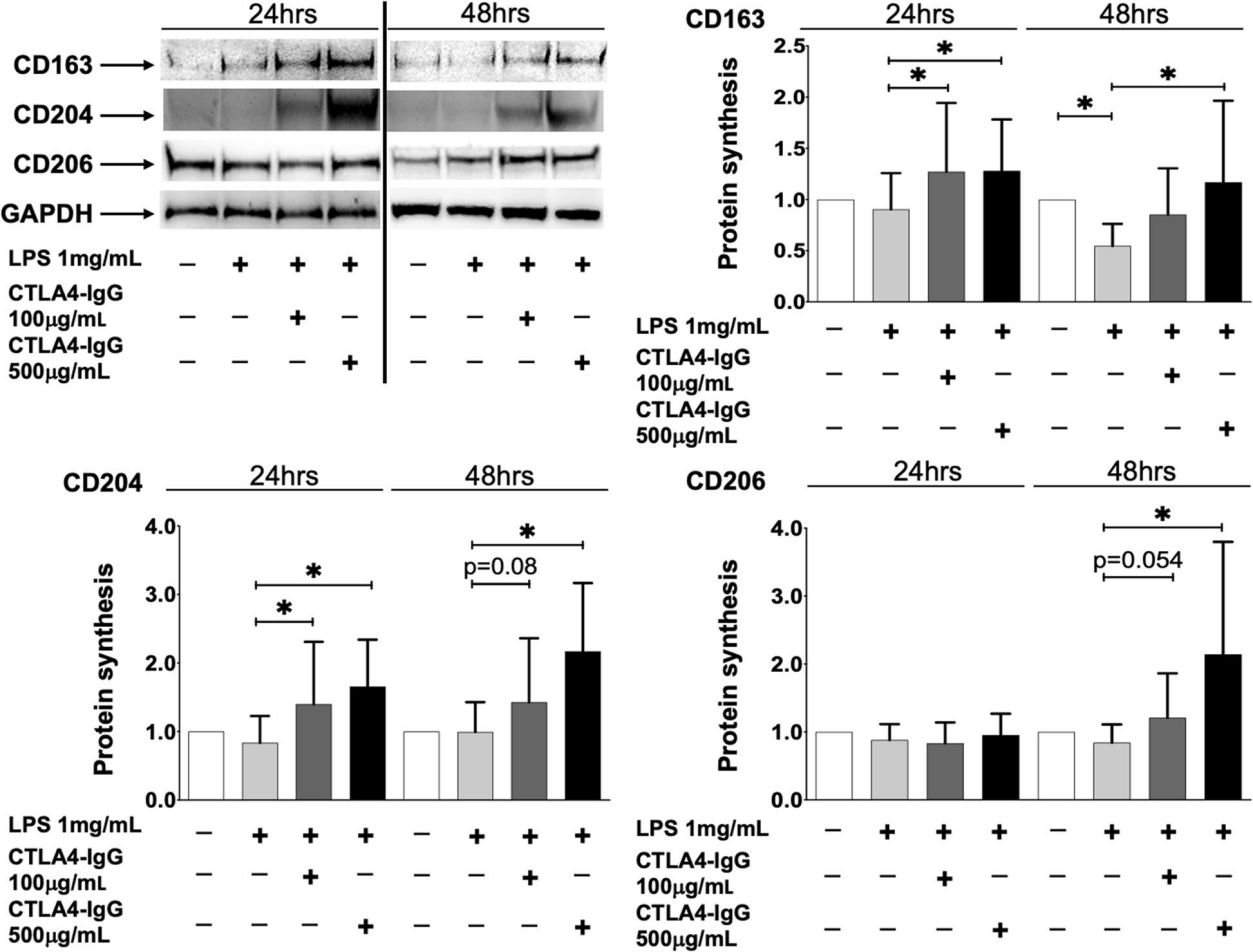
Protein synthesis of M2 phenotype markers in HS-M1-MDMs treated with CTLA4-Ig. Western blotting and related densitometric analysis of CD163, CD204, CD206, and GAPDH (HK gene) in cultured HS-MDMs maintained in normal growth medium without any stimulation or CTLA4-Ig treatment (white bar), stimulated with LPS alone (HS-M1-MDMs) (light grey bar), or stimulated with LPS and then treated with CTLA4-Ig (abatacept) at a concentration of 100 μg/mL (dark grey bar) and 500 μg/mL (black bar) for 24 and 48 h. The final results were obtained from ten independent in vitro experiments. *p < 0.05

LPS determined a slight decrease (not significant) in the synthesis of CD163, CD204, and CD206 after 24 h of stimulation compared to unstimulated cells; interestingly, a statistically significant reduction of CD163 after 48 h of stimulation was observed, suggesting a lag time for the protein synthesis (*p* < 0.05; Fig. 2).

Noteworthy, in cultured HS-M1-MDMs, both concentrations of CTLA4-Ig significantly blocked the LPS-induced decrease in CD163 and CD204 protein synthesis after 24 h of treatment, promoting an increase in their synthesis (*p* < 0.05 vs. untreated HS-M1-MDMs; Fig. 2). The synthesis of CD206 seems not to be further significantly modulated by the treatment with CTLA4-Ig (Fig. 2).

However, after 48 h of treatment, CTLA4-Ig significantly increased the protein synthesis of all the investigated M2 phenotype markers compared to untreated HS-M1-MDMs but limited to the high tested concentration (*p* < 0.05; Fig. 2).

To determine whether in cultured HS-M1-MDMs the effect of CTLA4-Ig was dose-dependent, the Spearman rank non-parametric correlation test was used. In cultured HS-M1-MDMs, the gene expression of TLR4 and CD80 after 3 h of treatment showed a “moderate” and statistically significant negative association with the CTLA4-Ig dosage as well as that of TLR4 and CD86 after 12 h of treatment (Table 2 A). As for the M2 markers, a statistically significant “strong” association between the dose-dependent effect of CTLA4-Ig and the gene expression of CD204 after 3 h of treatment was observed (rho = 0.72, *p* < 0.0001; Table 2 A). As for CD163 and CD206, a “weak” association (not significant) after 3 h and a significant “moderate” association after 12 h of treatment were found (Table 2 A). The results concerning the protein synthesis showed a “moderate” and statistically significant association for CD204 and CD163 after 24 h of treatment (Table 2 A). Furthermore, after 48 h of treatment, all M2 markers indicate a “moderate” statistically significant association with CTLA4-Ig dosage (Table 2 A).

**Table 2:**
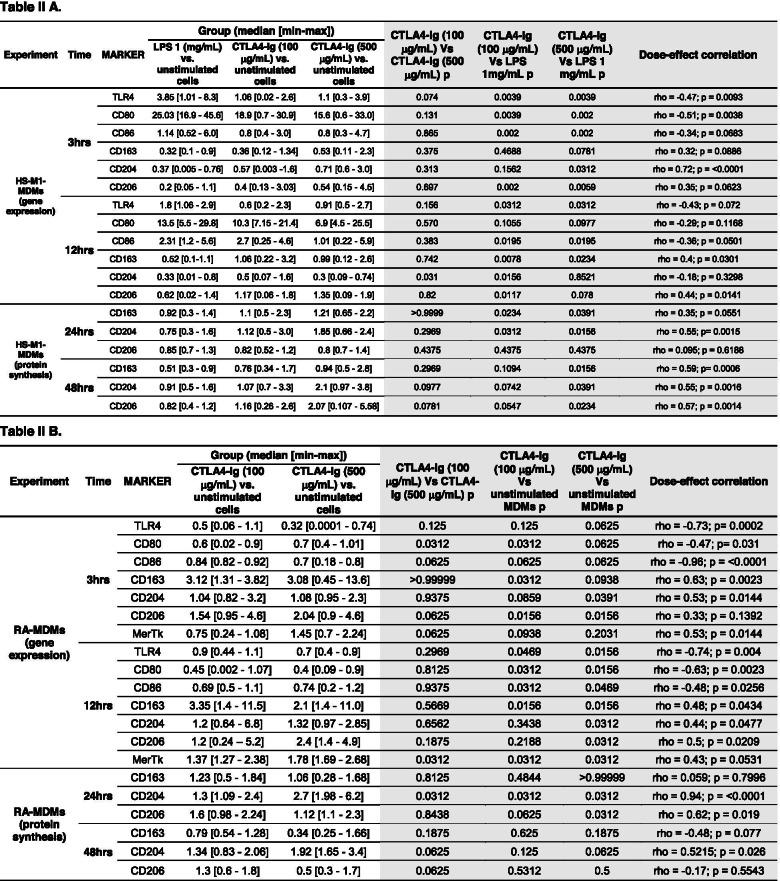
*P*-values of the gene expression of M1 and M2 markers in cultured HS-M1-MDMS and RA-MDMs and correlation statistical testing

The median (with range) of the gene expression and protein synthesis of M1 (TLR4, CD80, CD86) and M2 markers (CD163, CD204, CD206, MerTK) (A) in cultured HS-M1-MDMs and (B) in cultured RA-MDMs. In the white part of Table 2A, the gene expression and the protein synthesis correspond respectively to the expression and protein level (fold increase) of the target (gene or protein) in cultured HS-MDMs stimulated with LPS alone and stimulated with LPS followed by treatment with CTLA4-Ig compared with that of unstimulated HS-MDMs taken as the unit value by definition [43]. In the white part of Table 2 B, the gene expression and the protein synthesis correspond respectively to the expression and protein level (fold increase) of the target (gene or protein) in cultured CTLA4-Ig-treated RA-MDMs compared with that of untreated RA-MDMs taken as the unit value by definition [43]. In the light grey part of Table 2 A and B, the *p*-values related to the comparison between the experimental conditions and those related to the Spearman rank non-parametric correlation test were reported

### Abatacept significantly downregulated the TLR4 and CD80 M1 markers and upregulated the M2 marker in cultured RA-MDMs

The effect of the treatment with CTLA4-Ig in inducing an M2 phenotype was also investigated in cultured MDMs obtained from RA patients.

The preliminary results showed that cultured RA-MDMs were characterised by a higher basal gene expression of TLR4, CD80, and CD86 compared to cultured untreated HS-MDMs, confirming their activated M1 phenotype (Fig. S4). Based on these data, cultured RA-MDMs were not stimulated with LPS to further induce the pro-inflammatory M1 phenotype (already existing), but they were directly treated with CTLA4-Ig at the concentrations of 100 μM and 500 μM.

**Fig. 3:**
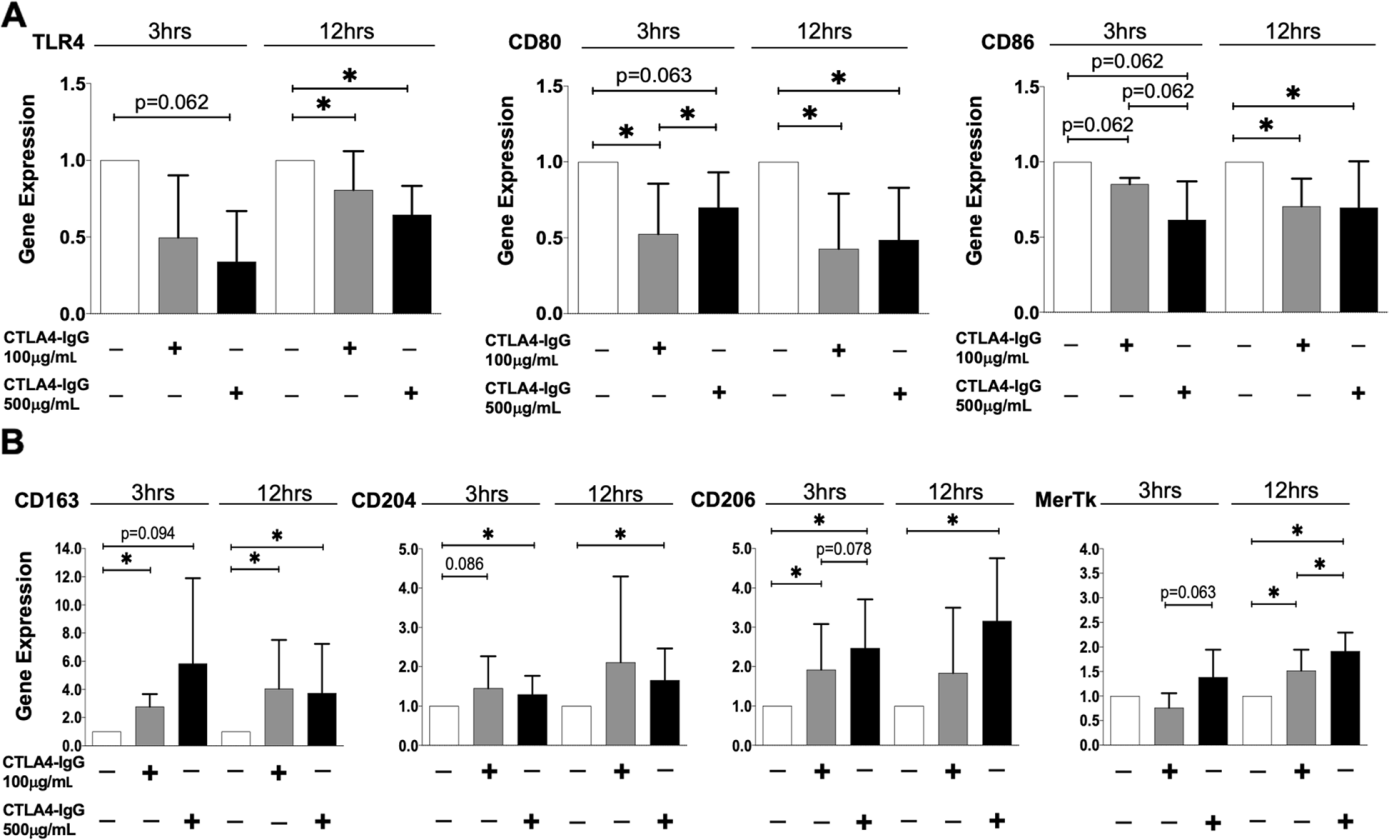
Gene expression of M1 and M2 markers in RA-MDMs treated with CTLA4-Ig. Quantitative real-time PCR of M1 (TLR4, CD80, CD86) or M2 (CD163, CD204, CD206, MerTK) markers in cultured human RA-MDMs maintained in normal growth medium without (white bar) or with CTLA4-Ig (abatacept) treatment at a concentration of 100 μg/mL (dark grey bar) and 500 μg/mL (black bar) for 3 and 12 h. **A** M1 markers (TLR4, CD80, CD86). **A** M2 markers (CD163, CD204, CD206, MerTK). The final results were obtained from 7 independent in vitro experiments. *p < 0.05

Therefore, in these cultured RA-MDMs, the treatment with both concentrations of CTLA4-Ig downregulated all the investigated M1 markers at both timings (Fig. 3A). Of note, CTLA4-Ig 100 μg/mL downregulated the gene expression of TLR4, CD86, and significantly the one of CD80 after 3 h of treatment compared to unstimulated RA-MDMs (CD80: *p* < 0.05), whereas the high tested concentration of CTLA4-Ig downregulated the gene expression of all M1 markers, but not in a statistically significant manner (*p* = 0.06 for all M1 markers) (Fig. 3A). Both concentrations of CTLA4-Ig significantly downregulated the gene expression of all tested M1 markers after 12 h of treatment compared to unstimulated RA-MDMs (*p* < 0.05 for all markers and for both concentrations; Fig. 3A). At the same time, the treatment with CTLA4-Ig induced an upregulation of the gene expression of all the surface M2 markers CD163, CD204, and CD206 (Fig. 3B). In particular, CTLA4-Ig 100 μg/mL significantly upregulated the gene expression of CD163 and CD206 after 3 h of treatment compared to unstimulated RA-MDMs (*p* < 0.05; Fig. 3B). The CD163 gene expression was maintained significantly increased by CTLA4-Ig 100 μg/mL also after 12 h of treatment (*p* < 0.05 vs. unstimulated cells; Fig. 3B). The high concentration of CTLA4-Ig upregulated the gene expression of CD204 and CD206 after both 3 and 12 h of treatment and that of CD163 only after 12 h of treatment compared to unstimulated RA-MDMs (CD204 and CD206: *p* < 0.05 at both 3 and 12 h; CD163: *p* < 0.05 at 12 h; Fig 3B).

In addition to the evaluation of surface M2 markers, the capability of CTLA4-Ig treatment to induce the upregulation of the gene expression of MerTK, a functional M2 marker relevant in RA pathogenesis, was also investigated in cultured RA-MDMs. In these cultured cells, both concentrations of CTLA4-Ig induced a significant upregulation of MerTK gene expression compared to unstimulated RA-MDMs, primarily after 12 h of treatment (*p* < 0.05 for both concentrations; Fig. 3B).

**Fig. 4:**
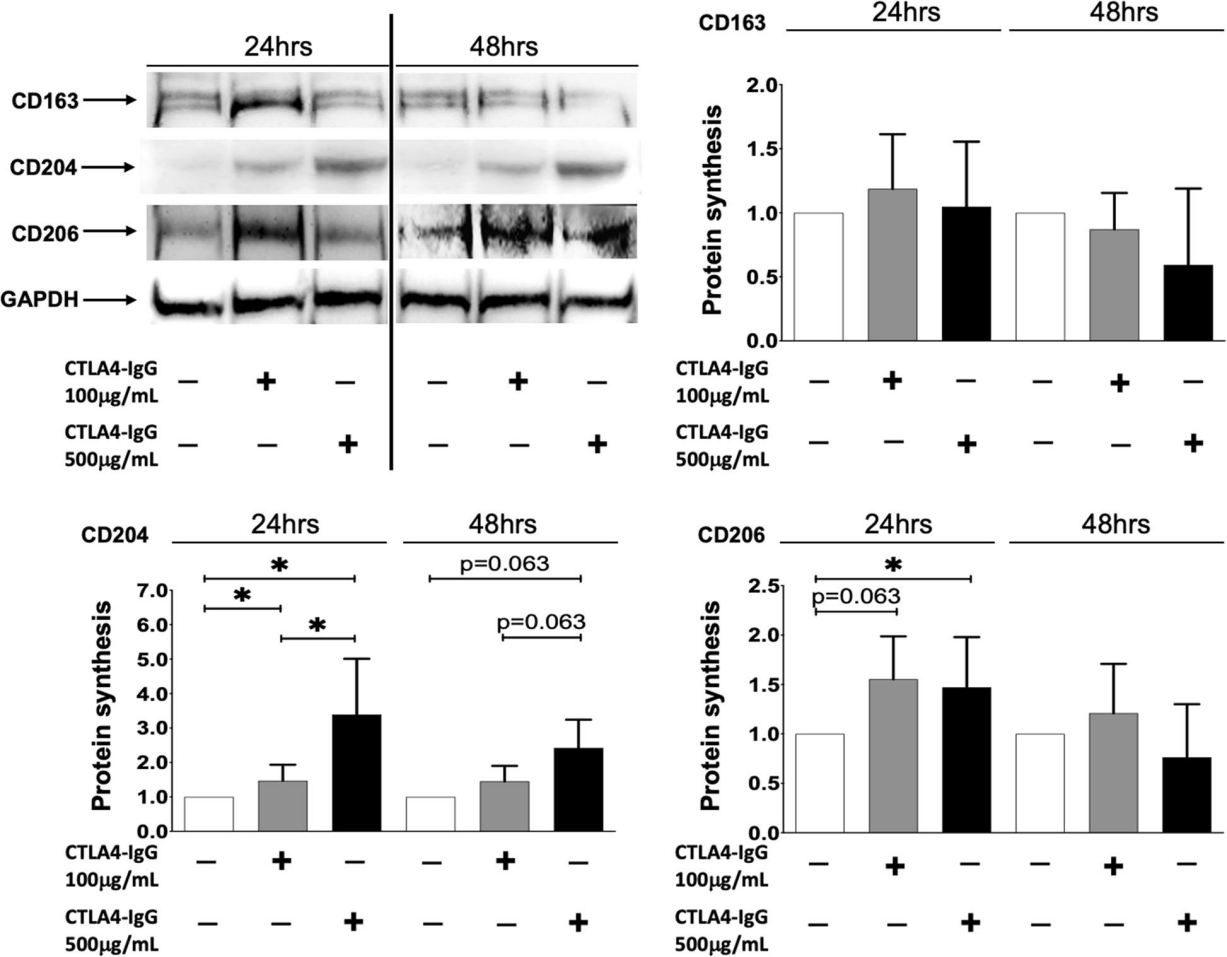
Protein synthesis of M2 phenotype markers in RA-MDMs treated with CTLA4-Ig. Western blotting and related densitometric analysis of CD163, CD204, CD206, and GAPDH in cultured RA-MDMs maintained in normal growth medium without (white bar) or with CTLA4-Ig (abatacept) treatment at a concentration of 100 μg/mL (dark grey bar) and 500 μg/mL (black bar) for 24 and 48 h. The final results were obtained from seven independent in vitro experiments

At the protein level, in cultured RA-MDMs, CTLA4-Ig 100 μg/mL slightly increased the synthesis of CD163 after 24 h of treatment, whereas the high concentration did not induce any upregulation of this M2 phenotype marker, even after 48 h of treatment compared to unstimulated cells (Fig. 4). Nonetheless, both concentrations of CTLA4-Ig induced a significant increase in the synthesis of CD204, primarily after 24 h of treatment compared to unstimulated RA-MDMs (*p* < 0.05 for both concentrations; Fig. 4). Moreover, CTLA4-Ig induced an increase in CD206 protein synthesis after 24 h of treatment, which was significant after treatment with the high concentration (*p* < 0.05 vs. unstimulated RA-MDMs; Fig. 4).

However, in cultured RA-MDMs, the Spearman non-parametric correlation test showed a significant “strong” negative association between the increase of the CTLA4-Ig dosage and the downregulation of the gene expression of TLR4 after 3 and 12 h of treatment and CD86 after 3 h of treatment (TLR4 rho = − 0.73, *p* = 0.0002; CD86 rho = − 0.96, *p* < 0.0001 after 3 h; TLR4 rho = − 0.74; *p* = 0.004 after 12 h), whereas the gene expression of CD80 (after 3 and 12 h of treatment) and CD86 (after 12 h of treatment) showed a significant “moderate” association with CTLA4-Ig dosage. In addition, the gene expression of all the M2 markers showed a significant “moderate” positive association with the dosage of CTLA4-Ig (Table 2 B). At the protein level, a significant “strong”/“moderate” (CD204 and CD206) positive association with CTLA4-Ig dosage was observed after 24 h of treatment (CD204 rho = 0.94, *p* < 0.0001; Table 2 B).

## Discussion

The results of the present study show that CTLA4-Ig treatment seems to promote the shift into an M2 phenotype of both cultured HS-M1-MDMs polarised by LPS stimulation and cultured MDMs obtained from RA patients. This shift was detected by the downregulation of the gene expression of M1 phenotype markers (TLR4, CD80, CD86) along with the upregulation of the gene expression of M2 phenotype markers (CD163, CD204, CD206) and their related protein synthesis. Of note, together with the upregulation of specific surface markers of M2 phenotype, the effect of CTLA4-Ig treatment in promoting the M1-M2 shift was observed also at the functional level, through the upregulation of gene expression of MerTK, which seems to play important functions potentially relevant in RA, as recently described [46].

In the pathogenesis of RA and joint damage, monocytes/macrophages are considered to play a central role, including newly differentiated peripheral blood monocytes, compared to fibroblasts, lymphocytes and neutrophils, which cooperate with different functions [7]. In fact, macrophages generate an inflammatory milieu by producing pro-inflammatory cytokines and chemotactic mediators, which further facilitate the synovial tissue invasion of other immune cells [29].

Although the ability of macrophages to assume various polarising profiles including the well-defined pro-inflammatory M1 and alternative M2 polarised phenotypes is frequently observed, an increased frequency of inflammatory M1 macrophages seems to mainly characterise RA patients with high disease activity compared to RA patients under remission, who conversely show an M2 phenotype prevalence [47, 48]. Several studies highlighted the important pathogenic role of M1 monocytes/macrophages in RA. In particular, the PBMCs of RA patients show an aberrant expression of CD14 and a high expression of CD86 (M1 marker) compared to that of HS monocytes, and they are suggested to play an important role in the pathophysiological processes of the disease [48]. Furthermore, Yoon et al. found that monocytes invading the synovial tissue express higher levels of CD80 and TLR4 than peripheral blood monocytes [17]. Moreover, a recent study highlighted that the PBMCs of RA patients express several M1 macrophage molecules, including class II major histocompatibility complex molecules (HLA-DR), CD64, CD86, and CCR5, while in the synovial fluid, they show a high level of HLA-DR, CD40, CD80, CD86, and CD276 [14].

A widely known in vitro model of RA inflammation and M1 phenotype induction is represented by the stimulation of monocytes (both THP1 cell line and peripheral blood monocytes) with LPS [22, 49]. Of note, LPS is able to induce a strong upregulation of CD80 and CD86 costimulatory molecules (among M1 markers), even though it requires other binding partners to induce a robust expression of TLR4 [50, 51]. In accordance with these observations, in our study, the upregulation of the gene expression of TLR4, CD80, and CD86 and the downregulation of that of CD163, CD204, and CD206 following LPS stimulation confirmed its ability to induce a M1 phenotype in cultured human HS-MDMs.

Our data are in line with those described by Degboe et al., although in Degboe’s study, cultured HS-MDMs were stimulated with LPS in combination with IFN-γ instead of LPS alone. However, in these cultured HS-MDMs stimulated with LPS+IFN-γ, the upregulation of CD80 and the downregulation of CD163 and CD206 gene expression were observed [29]. Although the stimulation of HS-MDMs used in our study and in Degboe’s study is different, the resulting induction of the polarisation into an M1 phenotype in these cultured cells seems to be very similar. Nevertheless, it is necessary to highlight that the LPS-induced polarisation of cultured HS-MDMs into a pro-inflammatory M1 phenotype represents an in vitro model, which cannot reflect the pathophysiological activity of RA macrophages. Based on this, to increase the clinical relevance of our study, the MDMs obtained from RA patients have been investigated.

It is noteworthy that monocytes/macrophages may contribute to the T cell activation and expansion during the inflammatory phase, thanks to their expression of HLA-DR and costimulatory molecules (CD80 and CD86), confirming their further functional role as antigen-presenting cells (APCs) [52]. In particular, to fulfil the T cell-naïve activation, the induction of CD80/86-CD28 costimulation is needed in these APCs. This T cell-naïve activation process is inhibited by the binding of CTLA-4 to CD80/86 [52, 53].

The CTLA4-Ig fusion protein (abatacept) is a bDMARD known to modulate RA inflammation, blocking T cell costimulation, and its effects on different cell types, involved in the immune-inflammatory reaction, have been widely analysed and demonstrated [31, 54]. Our previous studies highlighted the efficacy of CTLA4-Ig treatment to reduce the in vitro pro-inflammatory cytokine production (IL-6, TNFα, IL-1β) as well as the CD86 expression in cultured synovial macrophages, and this effect seems to be mediated by the upregulation of the NF-kB inhibitor IkB-a [36–38]. Moreover, Rochman et al. demonstrated the effect of abatacept in reducing the gene expression of NF-kB and AP-1 transcription factors along with the remarkable proliferation decrease of T regulatory cells [55]. Additionally, Lorenzetti et al. reported a dose-dependent decrease of CD80 and CD86 induced by the treatment with abatacept in B cells [56]. Lastly, abatacept is involved in the synovial cell infiltration regression [35, 57].

Since all these observations highlight the role of abatacept in reducing the inflammatory process, the present study investigated if this anti-inflammatory effect might be linked to its ability to promote an in vitro induction of the shift from an M1 to an M2 phenotype in cultured HS-M1-MDMs and RA-MDMs. However, current data regarding the in vitro activity of bDMARD to induce the shift from M1 to M2 macrophages are limited. Recently, Degboe et al. demonstrated the ability of the anti-TNFα bDMARDs to decrease CD80 and to increase CD163 and MerTK protein synthesis in cultured polarised M1-MDMs, whereas anti-IL-6 receptor or anti-CD20 agents (such as tocilizumab and rituximab) did not induce any upregulation of M2 phenotype markers [29]. The fact that anti-IL-6 receptor agents may not affect macrophages as well as their shift into an M2 polarised status is also confirmed by Chatzidionysiou’s study, in which it was demonstrated that no reduction in the number of macrophages was detectable in the synovial tissue samples of RA patients treated with tocilizumab [58].

The results of our study show that CTLA4-Ig treatment increased surface M2 phenotype markers (CD163, CD206, and CD204) in both cultured HS-M1-MDMs and RA-MDMs, at both gene and protein level (12 and 24 h, respectively), with variable statistically significant results. Interestingly, CTLA4-Ig treatment seems to induce a remarkable reduction in the gene expression of M1 markers (CD80, CD86, and TLR4) already at early stage (3 h), in distinct statistically significant manner.

Therefore, our observations may suggest a rapid onset of CTLA4-Ig action on the downregulation of M1 gene expression, remaining significantly stable over time (12 h), and followed by a clear induction of M2 phenotype that might confirm the M1–M2 shift. Noteworthy, CTLA4-Ig treatment already at low dosage (100 μM) seems to fulfil its maximum pharmacological action on M1 and M2 gene expression.

It should be considered that there are few “strong” monotone associations between CTLA4-Ig dosages and their modulatory effect on gene expression and protein synthesis of M1 and M2 markers in both cultured HS-M1-MDMs and RA-MDMs. It means that the gene expression or the protein synthesis (as dependent variable) always decrease (M1) or increase (M2) with the rising CTLA4-Ig dosages (as independent variable). The presence of moderate or weak associations might support the hypothesis that abatacept at early dosage (100 μM) already induce the gene modulation observed in this in vitro study.

Several studies confirmed that M2 macrophages are necessary for the correct resolution of inflammation, suggesting that the induction of shift from M1 to M2 phenotype can block the progression of many rheumatic diseases, including RA [19, 47]. The upregulation of CD163 on monocytes/macrophages surface expression induced by CTLA4-Ig in our experiments may suggest that the treatment could accelerate the maturation process towards the M2 phenotype reverting the M1/M2 disequilibrium which characterises the chronic inflammation of RA.

Furthermore, the concomitant increase in gene expression and protein synthesis of CD204 and CD206 might suggest that CTLA4-Ig treatment is able to induce the shift from M1 to all well-characterised subsets of M2 macrophages compared to other bDMARDs like anti-TNF-α bDMARDs [29]. They in fact seem to primarily promote the shift to an M2c macrophage subset, by inducing CD163 and MerTK expression through the increased production of IL-10, but not CD204 and CD206 expression, which is mediated by IL-4 instead [29]. In our in vitro study, the capability of CTLA4-Ig treatment to induce the upregulation of CD163 and MerTK gene expression, together with that of CD204 and CD206, in cultured RA-MDMs seems to promote the shift into an M2 macrophages phenotype which might have a role in the reduction of inflammation in RA patients. In support of this statement, an impressive rise in the presence of CD163 and CD206 markers on synovial tissue macrophages, together with MerTK, has been reported in RA patients in clinical remission [46]. As already mentioned, between M1 and M2, several other “M2-like” subtypes are observed; in fact, in other autoimmune connective tissue diseases, such as systemic sclerosis, circulating cells expressing both M1 and M2 phenotype markers were observed [59, 60]. In particular, CD163 is a scavenger receptor involved in haemoglobin-haptoglobin (HbHp) complex clearance leading to the release of IL-10 and carbon oxide (CO), which in turn exhibit strong anti-inflammatory activities [61, 62].

Besides a role in the resolution of inflammation, CD163 may also affect the initial steps of an adaptive immune response [7]. In fact, the M2 marker CD163 has been demonstrated to be involved in the adherence of human monocytes to endothelium, as well as in the inhibition of T lymphocyte proliferation in vitro [63, 64]. Moreover, in an ex vivo study by Ambarus et al., the presence of a cluster of CD163+ macrophages was identified in the intima layer of synovial tissue compared to the vascular/fibrous synovial layer [65].

Therefore, the shift towards CD163-expressing macrophages induced with abatacept might represent an additional aspect of its anti-inflammatory activity to attenuate inflammation in RA.

Some limitations characterise the present study. For example, even if the modulation of specific surface markers induced by CTLA4-Ig is important for the assessment of the shift from M1 towards M2 macrophages in RA, the evaluation of cytokines/chemokines produced by the M2 macrophages was not tested, to complete the evaluation, and must deserve further analysis. In addition, the less evident in vitro reactivity to the CTLA-4-Ig treatment for the RA-MDMs compared to the HS-M1-MDMs, might be partially due to the concomitant treatment of the RA patients with low-dose prednisone and/or csDMARDs [66]. However, ethical reasons justified the inclusion criteria and the concomitant minimal treatment. Finally, the limited number of RA patients analysed was due to the need to select patients with similar clinical and therapeutical characteristics.

Although this study demonstrated the in vitro capability of CTLA4-Ig to promote the shift from an M1 to an M2 phenotype in RA-MDMs, future experiments on cultured synovial macrophages need to be considered in order to improve the relevance of these results and the potential therapeutical importance of CTLA4-Ig treatment in RA patients.

Nevertheless, based on a higher effect of the high tested CTLA4-Ig concentration (500 μg/mL) in increasing the protein synthesis of tested M2 markers observed in cultured HS-M1-MDMs at longer lasting period, further experiments are needed to confirm a possible dose-dependent effect also in cultured RA-MDMs. The results of the present in vitro study, together with the knowledges reported in previous studies by our group as well as other groups, suggest a contribution of CTLA4-Ig treatment in ameliorating the clinical aspect in RA patients through the downregulation of the pro-inflammatory status that is determined by the presence at peripheral and tissue level of M1 polarised monocytes/macrophages which cooperate in the high disease activity. The reduction of this inflammatory status exerted by CTLA4-Ig in RA macrophages seems to occur through a rapid downregulation of specific markers of M1 phenotype (CD80, CD86, and TLR4) and the reduction of specific pro-inflammatory cytokines (IL-6, IL-1β, and TNFα), followed by the upregulation of specific markers of anti-inflammatory M2 phenotype (CD163, CD204, CD206, and MerTK). Moreover, starting from the results observed in this study, a future perspective which certainly might improve the impact of CTLA4-Ig treatment should be to investigate the capability of CTLA4-Ig to induce the shift from M1 to M2 phenotype in macrophages isolated from RA patients resistant to a previous therapy with anti-TNF drugs as well as in macrophages isolated from patients characterised by genetic variability in CTLA4 (such as those with a single nucleotide polymorphism CTLA-4 rs231775) that was recently demonstrated to decrease the risk of RA [67]. Based on the results described in this in vitro study, the release of specific anti-inflammatory cytokines and chemokines in these cultured shifted RA-MDMs along with their capability to activate a T-helper 2 response will be another future perspective of this study, primarily focusing on MDMs and synovial macrophages of naïve RA patients.

## Conclusions

In conclusion, the results of this in vitro study seems to indicate that abatacept can induce the M1 to M2 shift in both cultured HS-M1-MDMs and in RA-MDMs, suggesting an additional mechanism for its modulation of the RA inflammatory process [36–38].

## Supplementary Information

**Additional file 1: Figure S1.** Gene expression of M1 and M2 markers in cultured THP1-derived macrophages stimulated with LPS and a combination of LPS and IFN-γ. Quantitative real time PCR of M1 markers (TLR4, CD80, CD86) or M2 markers (CD163, CD204, CD206) in cultured THP1-derived macrophages maintained in normal growth medium without any stimulation (white bar), stimulated with LPS alone (1mg/ml) (light grey bar) or stimulated with LPS (1mg/ml) in combination with interferon-γ (IFN-γ 20ng/ml) (black bar) for 3 and 12 hrs. (A) M1 markers (TLR4, CD80, CD86); (B) M2 markers (CD163, CD204, CD206). Final results were obtained from five independent in vitro experiments.

**Additional file 2: Figure S2.** Gene expression of M1 and M2 markers in cultured human monocyte-derived macrophages stimulated with LPS and a combination of LPS and IFN-γ. Quantitative real time PCR of M1 markers (TLR4, CD80, CD86) or M2 markers (CD163, CD204, CD206) in cultured monocyte-derived macrophages obtained from healthy subjects and maintained in normal growth medium without any stimulation (white bar), stimulated with LPS alone (1mg/ml) (light grey bar) or stimulated with LPS (1mg/ml) in combination with interferon-γ (IFN-γ 20ng/ml) (black bar) for 3 and 12 hrs. (A) M1 markers (TLR4, CD80, CD86); (B) M2 markers (CD163, CD204, CD206). Final results were obtained from five independent in vitro experiments.

**Additional file 3: Figure S3.** Timeline of the experimental design. Timeline of the experimental design planned for the stimulation and treatment of cultured cells used for the *in vitro* experiments. *Experimental design 1*: Monocytes were obtained from peripheral blood mononuclear cells isolated from healthy subjects (HS PBMCs) after adhesion of 24 hrs in cell growth medium (RPMI at 10% of fetal bovine serum) and removal of T and B cells. Then monocytes were stimulated with phorbol myristate acetate (PMA 5ng/ml) for 24 hrs to induce their differentiation into monocyte-derived macrophages (MDMs). Culture MDMs were maintained in growth medium without stimulation or treatment (unstimulated), stimulated with LPS (1mg/ml), stimulated with LPS (1mg/ml) for 4 hrs and then treated with CTLA4-Ig (100μg/mL), stimulated with LPS (1mg/ml) for 4 hrs and then treated with CTLA4-Ig (500μg/mL). *Experimental design 2*: Monocytes were obtained from peripheral blood mononuclear cells isolated from rheumatoid arthritis patients (RA PBMCs) after adhesion of 24 hrs in cell growth medium (RPMI at 10% of fetal bovine serum) and removal of T and B cells. Then monocytes were stimulated with phorbol myristate acetate (PMA 5ng/ml) for 24 hrs to induce their differentiation into monocyte-derived macrophages (MDMs). Culture MDMs were maintained in growth medium without stimulation or treatment (unstimulated), treated with CTLA4-Ig (100μg/mL), treated with CTLA4-Ig (500μg/mL). Gene expression was investigated after 3 and 12 hrs by quantitative real-time polymerase chain reaction, whereas protein synthesis was investigated by Western blotting (and related densitometric analysis) after 24 and 48 hrs of stimulation and treatment.

**Additional file 4: Figure S4.** Gene expression of M1 markers in cultured human monocyte-derived macrophages obtained from healthy subjects and RA patients. Quantitative real time PCR of M1 markers (TLR4, CD80, CD86) in cultured monocyte-derived macrophages (MDMs) obtained from healthy subjects (white bar) and rheumatoid arthritis patents (black bar) maintained in normal growth medium without any stimulation for 3 and 12 hrs. Final results were obtained from five *independent in vitro* experiments.

## Data Availability

All data generated or analysed during this study are included in this published article [and its supplementary information files]. Data are available upon request to qualified investigators.

## Abbreviations

RA: Rheumatoid arthritis
M1: Classically activated macrophages
M2: Alternatively activated macrophages
CTLA4-IgG: Abatacept
HS: Healthy subjects
CD: Cluster differentiation
TLR: Toll-like receptors
MDMs: Monocyte-derived macrophages
HS-M1-MDMs: Healthy subjects’ M1 monocyte-derived macrophages
RA-MDMs: Rheumatoid arthritis patients’ monocyte-derived macrophages
MMPs: Matrix metalloproteinases
TNFa: Tumour necrosis factor-a
IL-1: Interleukine-1
CD204/CD163: Macrophage scavenger receptors
CD206: Mannose receptor 1
TGF-β: Transforming growth factor-β
GM-CSF: Granulocyte-macrophage colony-stimulating factor
M-CSF: Macrophage colony-stimulating factor
IFN-γ: Interferon-γ
LPS: Lipopolysaccharides
ACPA: Anticitrullinated protein antibodies
IRF: Interferon regulatory factor
bDMARD: Biological disease-modifying antirheumatic drugs
EULAR: European League Against Rheumatism
NF-kB: Nuclear factor kappa-light-chain-enhancer of activated B cells
PCR: Polymerase chain reaction
ACR: American College of Rheumatology
NSAID: Nonsteroidal anti-inflammatory drugs
csDMARDs: Conventional synthetic disease-modifying antirheumatic drugs
PBMCs: Peripheral blood mononuclear cells
RPMI: Roswell Park Memorial Institute
PMA: Phorbol myristate acetate
qRT-PCR: Quantitative real-time polymerase chain reaction
RNA: Ribonucleic acid
cDNA: Complementary deoxyribonucleic acid
MerTK: MER proto-oncogene, tyrosine kinase
HK: Housekeeping gene
GAPDH: Glyceraldehyde 3-phosphate dehydrogenase
b-Actin: Beta-actin
UB2D2: Ubiquitin-conjugating enzyme E2 D2
RPL13A: Ribosomal protein L13a
THP1: Human monocytic leukaemia
2^−ΔΔCT^: 2-Delta-delta cycle threshold
SYBR GREEN: (SG) asymmetrical cyanine dye
RIPA: Radio-immunoprecipitation assay
TrisHCl: Tris(hydroxymethyl)aminomethane-Hydrochloric acid
NaCl_2_: Sodium chloride
SDS: Sodium dodecyl sulphate
EGTA: Ethylene glycol-bis(β-aminoethyl ether)-N,N,N′,N′-tetraacetic acid
EDTA: Ethylenediaminetetraacetic
Na_3_VO_4_: Sodium orthovanadate
PMSF: Phenylmethylsulphonyl fluoride
BCA: Bicinchoninic acid
PBS: Phosphate-buffered saline
BSA: Bovine serum albumin
HRP: Horseradish peroxidase
S.D.: Standard deviation
FCgRIII: Fragment crystallizable g receptor III
HLA-DR: Human leukocyte antigen–DR isotype
APC: Antigen-presenting capacity
CTLA-4: Cytotoxic T lymphocyte-associated protein 4
IkB-a: Nuclear factor of kappa light polypeptide gene enhancer in B cell inhibitor alpha
