# Supplementary figures and images for: CTLA4-Ig treatment induces M1–M2 shift in cultured monocyte-derived macrophages from healthy subjects and rheumatoid arthritis patients
Source: Arthritis Res Ther. 2021 Dec 24;23:306. doi: 10.1186/s13075-021-02691-9 (PMC8709961; doi:10.1186/s13075-021-02691-9)

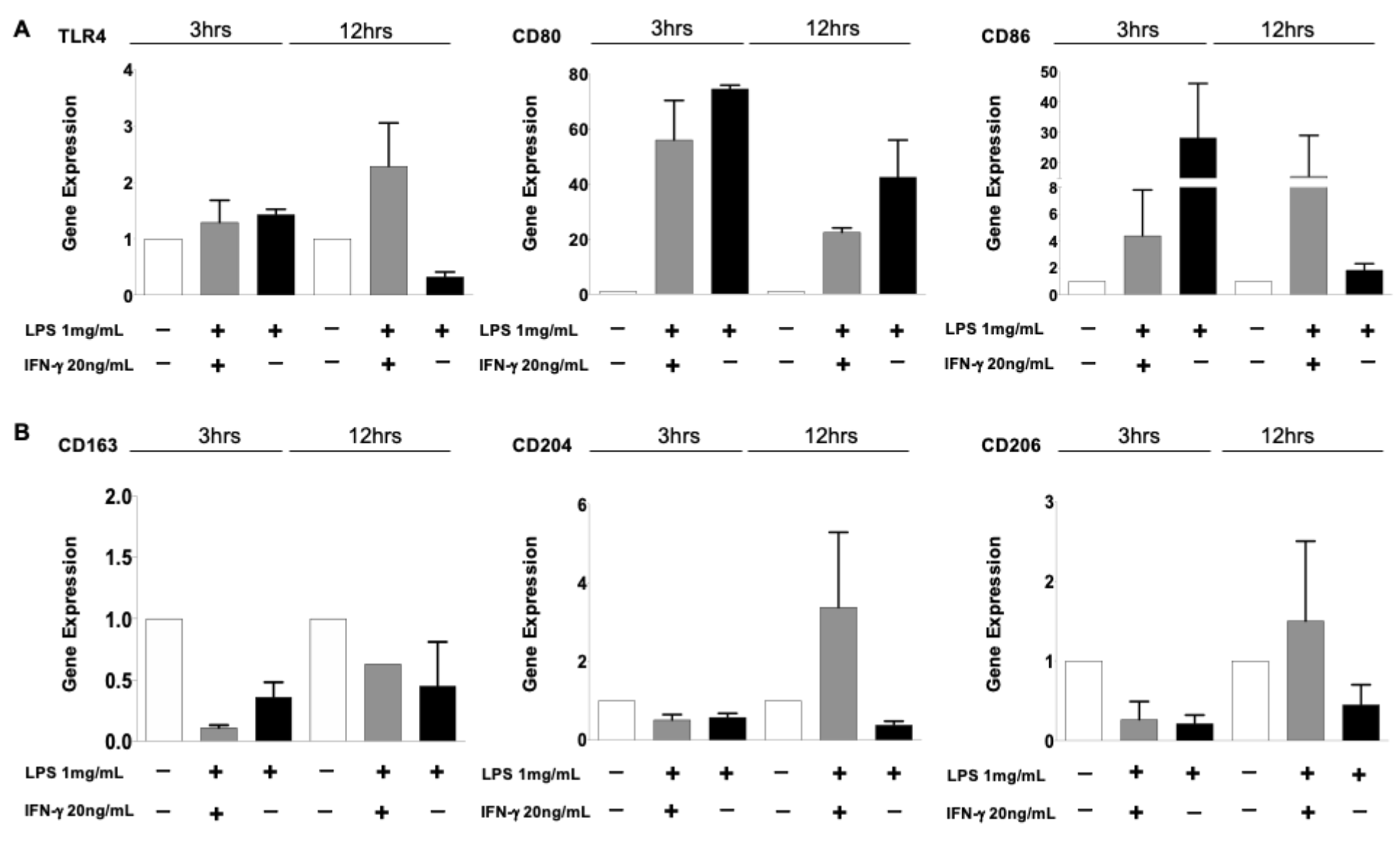

Supplement: Supplementary file 1 — Additional file 1: Figure S1. Gene expression of M1 and M2 markers in cultured THP1-derived macrophages stimulated with LPS and a combination of LPS and IFN-γ. Quantitative real time PCR of M1 markers (TLR4, CD80, CD86) or M2 markers (CD163, CD204, CD206) in cultured THP1-derived macrophages maintained in normal growth medium without any stimulation (white bar), stimulated with LPS alone (1mg/ml) (light grey bar) or stimulated with LPS (1mg/ml) in combination with interferon-γ (IFN-γ 20ng/ml) (black bar) for 3 and 12 hrs. (A) M1 markers (TLR4, CD80, CD86); (B) M2 markers (CD163, CD204, CD206). Final results were obtained from five independent in vitro experiments. [file 13075_2021_2691_MOESM1_ESM.tiff]

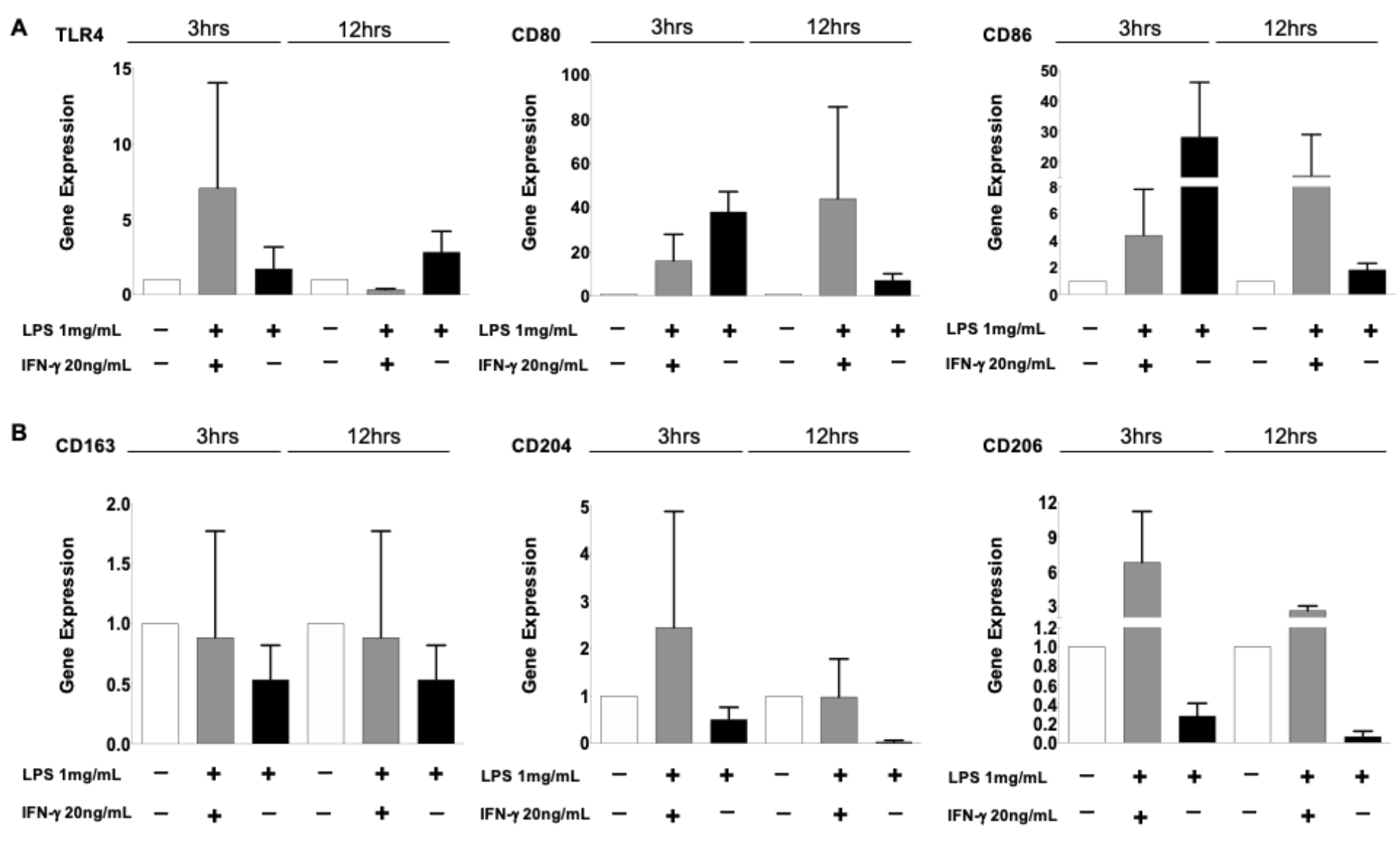

Supplement: Supplementary file 2 — Additional file 2: Figure S2. Gene expression of M1 and M2 markers in cultured human monocyte-derived macrophages stimulated with LPS and a combination of LPS and IFN-γ. Quantitative real time PCR of M1 markers (TLR4, CD80, CD86) or M2 markers (CD163, CD204, CD206) in cultured monocyte-derived macrophages obtained from healthy subjects and maintained in normal growth medium without any stimulation (white bar), stimulated with LPS alone (1mg/ml) (light grey bar) or stimulated with LPS (1mg/ml) in combination with interferon-γ (IFN-γ 20ng/ml) (black bar) for 3 and 12 hrs. (A) M1 markers (TLR4, CD80, CD86); (B) M2 markers (CD163, CD204, CD206). Final results were obtained from five independent in vitro experiments. [file 13075_2021_2691_MOESM2_ESM.tiff]

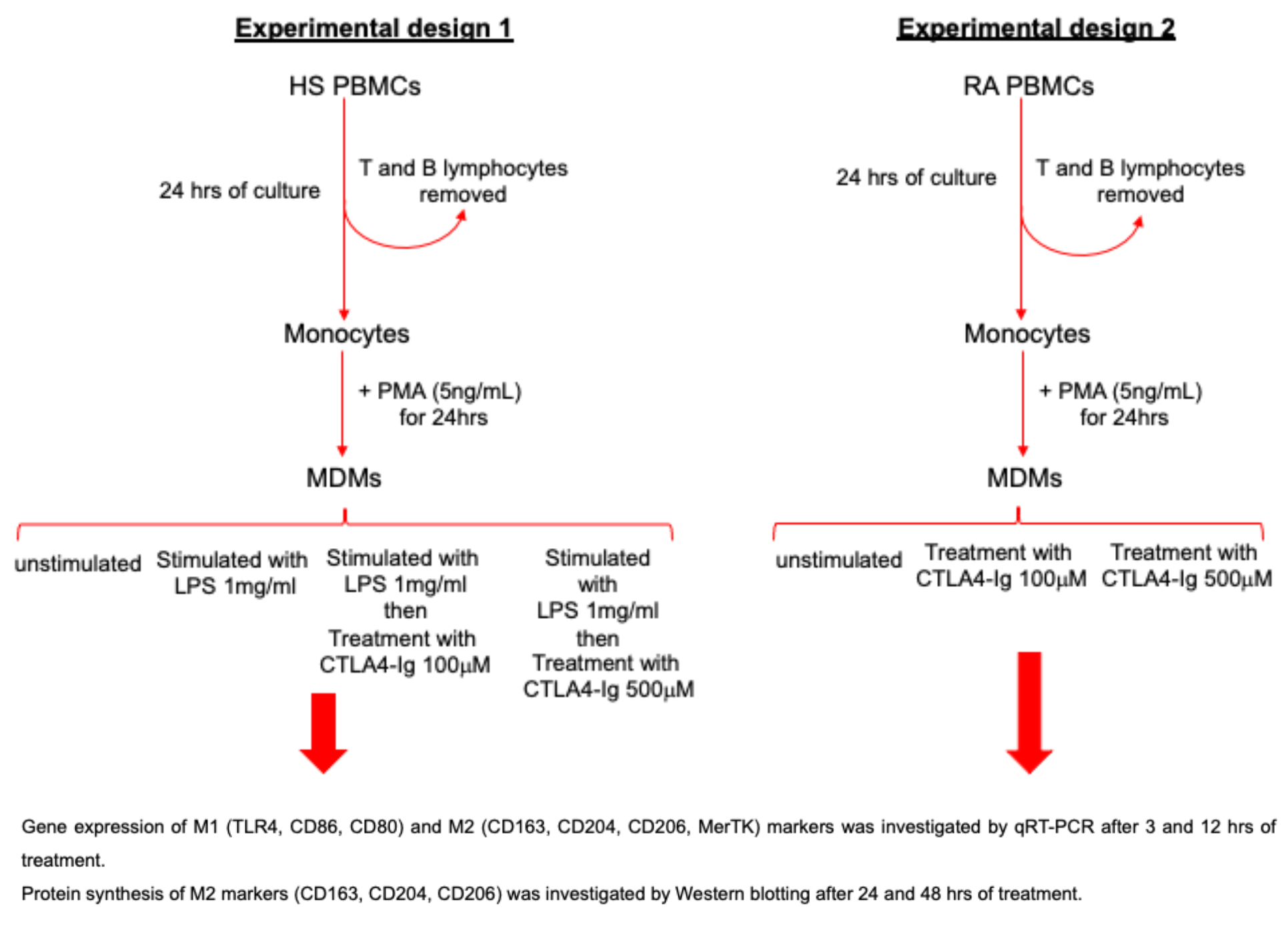

Supplement: Supplementary file 3 — Additional file 3: Figure S3. Timeline of the experimental design. Timeline of the experimental design planned for the stimulation and treatment of cultured cells used for the in vitro experiments. Experimental design 1: Monocytes were obtained from peripheral blood mononuclear cells isolated from healthy subjects (HS PBMCs) after adhesion of 24 hrs in cell growth medium (RPMI at 10% of fetal bovine serum) and removal of T and B cells. Then monocytes were stimulated with phorbol myristate acetate (PMA 5ng/ml) for 24 hrs to induce their differentiation into monocyte-derived macrophages (MDMs). Culture MDMs were maintained in growth medium without stimulation or treatment (unstimulated), stimulated with LPS (1mg/ml), stimulated with LPS (1mg/ml) for 4 hrs and then treated with CTLA4-Ig (100μg/mL), stimulated with LPS (1mg/ml) for 4 hrs and then treated with CTLA4-Ig (500μg/mL). Experimental design 2: Monocytes were obtained from peripheral blood mononuclear cells isolated from rheumatoid arthritis patients (RA PBMCs) after adhesion of 24 hrs in cell growth medium (RPMI at 10% of fetal bovine serum) and removal of T and B cells. Then monocytes were stimulated with phorbol myristate acetate (PMA 5ng/ml) for 24 hrs to induce their differentiation into monocyte-derived macrophages (MDMs). Culture MDMs were maintained in growth medium without stimulation or treatment (unstimulated), treated with CTLA4-Ig (100μg/mL), treated with CTLA4-Ig (500μg/mL). Gene expression was investigated after 3 and 12 hrs by quantitative real-time polymerase chain reaction, whereas protein synthesis was investigated by Western blotting (and related densitometric analysis) after 24 and 48 hrs of stimulation and treatment. [file 13075_2021_2691_MOESM3_ESM.tiff]

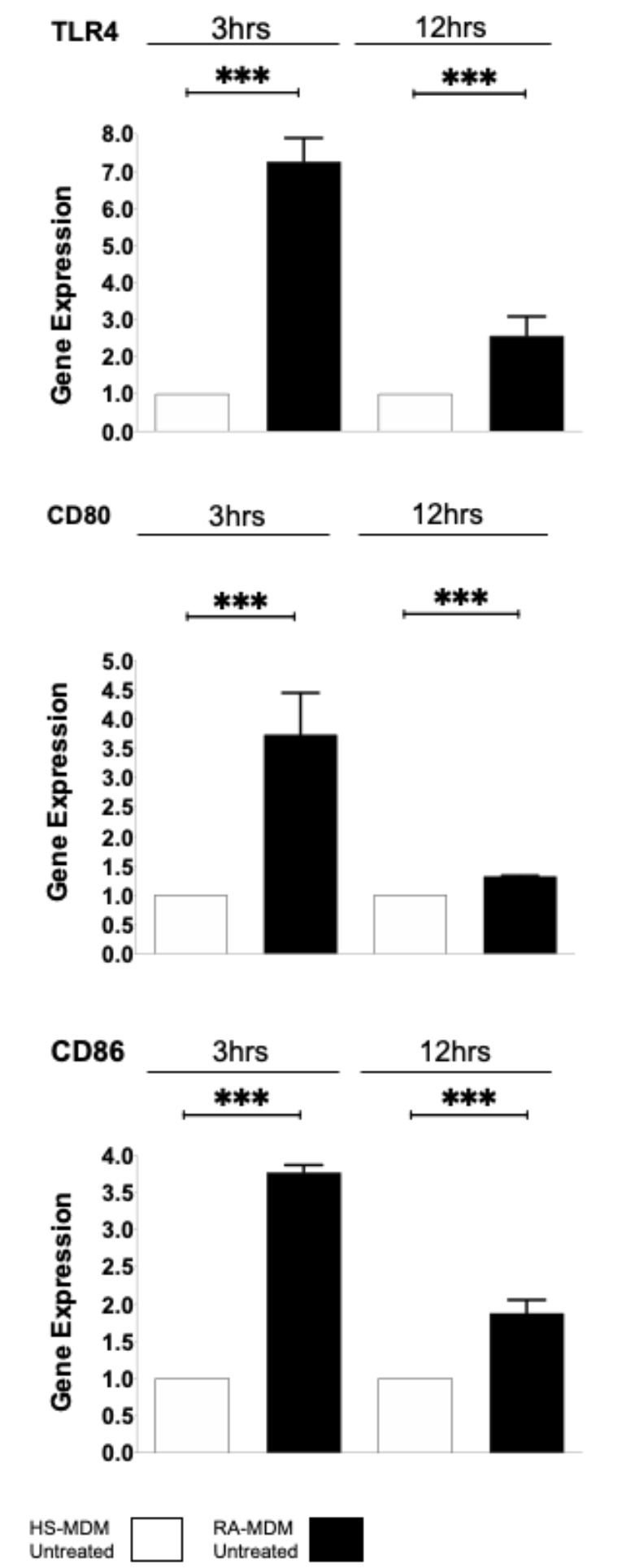

Supplement: Supplementary file 4 — Additional file 4: Figure S4. Gene expression of M1 markers in cultured human monocyte-derived macrophages obtained from healthy subjects and RA patients. Quantitative real time PCR of M1 markers (TLR4, CD80, CD86) in cultured monocyte-derived macrophages (MDMs) obtained from healthy subjects (white bar) and rheumatoid arthritis patents (black bar) maintained in normal growth medium without any stimulation for 3 and 12 hrs. Final results were obtained from five independent in vitro experiments. [file 13075_2021_2691_MOESM4_ESM.tiff]
